# Diseases of the Pancreas

**Published:** 1904-11-19

**Authors:** 


### PROGRESS IN MEDICINE AND SURGERY.

### DISEASES OF THE PANCREAS.

(*Concluded from page* 126.)

**Acute and Chronic Pancreatitis.**—Fat necrosis and hæmorrhages are, according to Opie,[14] most frequent in acute pancreatitis, less frequent in suppurative pancreatitis. Fat necrosis, however, may accompany chronic inflammations or obstruction of the pancreatic duct from various causes. Flexner[15] has shown that injections of sterile blood into the duct do not cause acute inflammation, although the injection of bile and other substances sets up acute pancreatitis with hæmorrhages. The latter he therefore believes not to be the cause of the inflammation but its sequel. Mayo Robson[16] holds that the hæmorrhages are sometimes part of a general hæmorrhagic tendency and are intensified by the presence of jaundice, though they may occur apart from either. In some cases, both acute and chronic, the hæmorrhages are absent, and in some purely local. He suggests that the glycerine set free by the fat necrosis may cause these, but Hess[17] finds that the injection of glycerine into the pancreatic duct in dogs is without effect, and ascribes the hæmorrhage and fat necrosis to the regurgitation of fatty acids from the intestine. Opie,[18] out of 41 cases in which acute hæmorrhagic pancreatitis was associated with gall-stones, found the common duct blocked by a stone in seven, and considers the regurgitation of bile to be the determining cause of the condition. The diagnosis may thus be assisted by the presence of jaundice or the history of attacks of gall-stone colic; Woolsey[19] dwells on the sudden onset with severe colicky epigastric pain, becoming generalised later, and the severe vomiting, evacuation, and distension with rigidity and tenderness over the upper abdomen. There are also frequent collapse and cyanosis, with a small, rapid pulse. The temperature is not high, in fact it is often subnormal. Alcoholism, corpulence, and a history of gall-stones, gastro-duodenal catarrh, or trauma make the diagnosis more probable. In many cases laparotomy alone can clear up the diagnosis, which he also recommends as the only possible treatment. Weir[20] lays stress on the presence of bloody fluid in the abdominal cavity as a valuable diagnostic sign of acute pancreatitis, though Libman does not consider it important unless of a chocolate colour; in a case of chronic pancreatitis recorded by Ehler,[23] a dark chocolate coloured fluid was found in a retention cyst in the tail of the pancreas. Woolsey and Weir regard the effusion into the peritoneum as very toxic and irritating. The former says it occasionally contains bacteria, and both agree that its early evacuation and drainage offer the best chance to the patient of recovery; of 46 cases operated on, however, only nine recovered. Chronic pancreatitis occurs in two forms according to Opie,[21] interlobular and intralobular. The former is most frequently associated with diabetes. The commonest cause of the condition is a gall-stone impacted in the common duct, and it may also occur by obstruction from pancreatic calculi, or carcinoma of the head of the pancreas. Without actual obstruction, an inflammatory condition commencimg in the duodenum, or bile duct, may extend to the pancreatic duct. All these tend to produce interlobular pancreatitis without glycosuria. The other form is associated with portal cirrhosis and arterio-sclerosis, and hyaline degeneration of the islands of Langerhans. In nine cases out of 11 there was glycosuria, and in the remaining two the islands were hardly affected at all. The symptoms of chronic pancreatitis are given by Mayo Robson[22] as (1) pain, sometimes gradual and sometimes acute in onset, localised in the middle of the epigastrium, and passing back between the shoulders or to the left. There is also a tender spot an inch above the navel in the middle line. (2) Jaundice usually at some time in the history of the case, becoming chronic. (3) Loss of strength and flesh, vomiting and diarrhœa, and fatty and offensive stools. (4) Albuminuria and glycosuria. (5) Fever occasionally. (6) Hæmorrhages from mucous surfaces or into the skin associated with jaundice. (7) Distension of the gall-bladder. These symptoms are well illustrated by a case recorded by Ehler,[23] which until the operation was considered to be one of gall-stones or carcinoma of the bile ducts. The patient died shortly after the operation, at which no gall-stones were found, but a hard lump was felt and considered to be carcinoma of the pancreas. Post-mortem there was chronic indurative pancreatitis, apparently due to changes in the vascular supply of the organ. Woolsey[24] sums up the symptoms in a similar manner, and notes the difficulty of diagnosis even after operation. With Mayo Robson he recommends direct drainage of the gall-bladder as completely curative. He notes, however, the danger of incision of the pancreas owing to the chances of hæmorrhage or leakage of the external secretion.

**Necrosis of the Pancreas.**—Cases illustrating this condition have been collected from the literature by Trevor.[25] In Peiser's case of complete necrosis of the entire organ the patient, a woman aged 28, died of severe diabetes four months after removal of the necrotic mass. The cause was apparently hæmorrhage into the pancreas subsequent to severe anæmia from *post-partum* flooding. In Selberg's case the necrosis followed a kick from a horse, and the symptoms pointed to perforative peritonitis. No operation was performed owing to the hopeless condition of the patient, and the correct diagnosis was only ascertained at the autopsy. Brentario's case survived seven years with severe diabetes.

**Carcinoma of the Pancreas.**—This is the commonest tumour of the pancreas, and Mayo Robson[26] details the symptoms as follows:—The patient, usually over 40 years of age, after suffering from "indigestion" for a time becomes jaundiced. The jaundice deepens, the gall-bladder is found distended, and the liver somewhat enlarged. A tumour in the pancreas may be felt. Cachexia and pain progress rapidly, and the fæces contain fat or fatty acids, and a large amount of undigested muscle fibre. Albuminuria is frequent, glycosuria and lipuria rare. The patient usually dies about six or eight months after the development of jaundice. The diagnosis from chronic pancreatitis is made from the short history, the extreme cachexia and the absolute jaundice with absence of a history of gall-stones. The presence of anasarca or ascites, and the absence of tenderness below the right costal margin, together with the position and characteristics of the tumour will render the diagnosis more certain. The ague-like attacks of infective cholangitis are against the diagnosis of cancer. Cancer of the pylorus is distinguished by the gastric symptoms, but may co-exist. Cancer of the common bile duct involving the papilla is rare, and cannot be distinguished from pancreatic carcinoma. Lauder Brunton[27] has recorded a case in which a nodule of carcinoma was felt near the pylorus, and in which the subsequent development of jaundice suggested the diagnosis of cancer of the head of the pancreas. Post-mortem, however, the pancreas was found free from growth, but the glands near the pancreas and in the hilum of the liver were affected and pressed on the common duct. He also gives details of a case in which the ordinary symptoms were present, but owing to a second pancreatic duct undigested meat fibres were not present in the fæces. There was no glycosuria. He concludes that though a positive diagnosis may often be correctly made, the possibility of cancer outside the pancreas must be remembered, and too much reliance must not be placed on symptoms arising from obstruction to the pancreatic secretion.

**Sarcoma of the Pancreas.**—This is considered undoubtedly rare as a primary condition by Mayo Robson,[28] though secondary cases are less uncommon. If situated in the tail of the organ he thinks it might be treated surgically, and quotes one successful case. Kakels,[29] however, finds that out of 21 cases of sarcoma only 10 were primary, and only four occurred in the tail of the organ, including one doubtful one. In Kakels' own case splenic tumour was excluded by an examination of the blood, and, owing to the presence of albuminuria, sarcoma of the kidney was diagnosed. Operation was found impossible. Post-mortem, a very vascular mixed-celled sarcoma was found associated with granular kidneys. Fawcett[30] has recorded a case in which a lympho-sarcoma involved the whole gland, but its origin was not absolutely clear. It was associated with suppurative peritonitis, severe suppurative gingivitis and pseudo-lipæmia. The latter condition was demonstrated from the heart's blood post-mortem. The blood separated into two layers like lipæmic blood, but contained no excess of fat. The urine contained neither sugar nor diacetic acid. The blood condition remained obscure both as to its chemistry and origin.

[14] Med. Rec., Feb. 20, 1904, p. 313. [15] Practitioner, April 1904, p. 590. [16] Ibid. [17] Ibid., p. 577. [18] Med. Rec., Feb. 20, 1904, p. 313. [19] Ibid. [20] Ibid., p. 313. [21] Practitioner, April, 1904, p. 581. [22] Ibid., p. 582. [23] Lancet, Feb. 13, 1904, p. 454. [24] Loc. cit. [25] Practitioner, April 1904, p. 579. [26] Brit. Med. Jour., April 2, 1904, p. 774. [27] Loc. cit. [28] Brit. Med. Jour., April 2, 1904, p. 775. [29] Practitioner, April 1904, p. 587. [30] Lancet, May 7, 1904, p. 1280.

